# Molecular Prevalence and Genotypic Diversity of *Theileria equi* in Xinjiang, China, Based on Three Genes

**DOI:** 10.3390/vetsci13010027

**Published:** 2025-12-25

**Authors:** Sinan Qin, Telieke Kulabieke, Duman Mizhamuhan, Mengyuan Zhang, Min Jin, Gulibositan Abula, Mengjie Pi, Haorui Wang, Yang Zhang, Qingyong Guo

**Affiliations:** 1 Parasitology Laboratory, College of Veterinary Medicine, Xinjiang Agricultural University, Urumqi 830052, China; 320232806@xjau.edu.cn (S.Q.); 320222740@xjau.edu.cn (M.J.); 320242797@xjau.edu.cn (G.A.); 320242815@xjau.edu.cn (M.P.); 320252846@stu.xjau.edu.cn (H.W.); 2 Animal Diseases Control and Prevention Centre of Xinjiang Uygur Autonomous Region, Urumqi 830011, China; tilek_vet@163.com; 3 Arele Town Agricultural Development Service Center, Qinghe 836200, China; 18034866262@163.com; 4 Hami City Animal Disease Prevention and Control Center, Hami 839000, China; 18399766804@163.com

**Keywords:** *Theileria equi*, epidemiology, genotype, haplotype

## Abstract

Equine theileriosis, caused by the protozoan parasite *Theileria equi*, represents a significant economic threat to the equine industry. In Xinjiang, China, an endemic area for this disease, systematic research on the genetic diversity of *T. equi* has been notably lacking. The aim of this study was to obtain molecular epidemiological data pertaining to the parasite in selected regions of Xinjiang, China, and analyze the genetic characteristics (including *18S* rRNA, *EMA-1*, and *COI* genes) and genotype distribution patterns of isolates from these regions, providing a scientific basis for developing targeted prevention and control strategies. Blood samples were collected from 440 horses across four regions (Altay, Ili, Tacheng, and Urumqi) and subjected to PCR assays. Positive samples were sequenced for phylogenetic and haplotype network analyses, and genetic diversity indices were calculated. The overall infection rate of *T. equi* was 38.41% (169/440), with Tacheng having the highest prevalence (86.27%) and Altay the lowest (20.88%); these regional differences were statistically significant. Phylogenetic analysis identified two genotypes of the *18S* rRNA gene: genotype E (predominant) and genotype A. All *EMA-1* sequences clustered exclusively within genotype A. Notably, all *COI* gene sequences formed a monophyletic group, exhibiting closer genetic relationships to isolates from France and Senegal. This study presents the first comprehensive genotyping of *T. equi* in Xinjiang based on three target genes and constructs an associated haplotype network. The analysis identified *18S* rRNA genotype E and *EMA-1* genotype A as the predominant genotypes. Furthermore, the genetic diversity of *T. equi* was found to be higher in Urumqi than in the other regions studied.

## Simple Summary

Equine theileriosis, a disease caused by the protozoan parasite *Theileria equi* and trans-mitted by ticks, represents a significant threat to equine health and the equine industry in Xinjiang, China. To investigate the current prevalence and genetic characteristics of the parasite, we conducted a molecular survey and genetic analysis. Blood samples were collected from 440 apparently healthy horses across four regions (Altay, Ili, Tacheng, and Urumqi). The overall infection rate was 38.41%, with prevalence varying significantly by region; it was highest in Tacheng (86.27%) and lowest in Altay (20.88%). Genetic characterization based on three target genes revealed the following: analysis of the *18S* rRNA gene identified two distinct genotypes (E and A), with genotype E being overwhelmingly dominant. All parasites tested belonged to genotype A for the *EMA-1* gene. For the mitochondrial *COI* gene, local strains showed a close phylogenetic relationship to isolates reported from France and Senegal. Haplotype network analysis indicated that Urumqi harbored the highest genetic diversity, suggesting it may be a hotspot for parasite evolution. This study provides the first integrated genetic dataset for *T. equi* in Xinjiang using three different molecular markers, offering crucial insights for monitoring and targeted control of this important disease.

## 1. Introduction

Equine piroplasmosis is a tick-borne disease that poses a significant economic burden on the equine industry worldwide. Except for a few countries, such as Japan, the United States, and Australia, where it has not been reported, the disease is prevalent in many other countries [1]. Equine piroplasmosis is caused by three pathogens: *Theileria equi* (formerly described as *Babesia equi*), *B*. *caballi*, and *T. haneyi* [2,3,4,5,6].

These parasites infect all equid species, including horses, donkeys, mules, and zebras; however, clinical disease is rare in donkeys, mules, and zebras, allowing the disease to spread more easily and posing a global threat to equine health and productivity [2,3,7,8,9]. Infection with *T. equi* and *B. caballi* produces similar clinical signs, including fever, inappetence, icterus, and, in some severe cases, even death; however, clinical presentation tends to be more severe in cases of *T. equi* infection [2,10,11,12]. In contrast, the recently identified *T. haneyi* is rarely associated with clinical signs, even in splenectomized horses [12,13]. Consequently, equine piroplasmosis not only reduces the production performance of equine animals but also leads to increased veterinary costs, expenses for tick control, and restrictions on animal movement [2,14]. As an example, the joint APHIS-VS and Florida *B. caballi* eradication program took 25 years and cost USD 12 million for tick inspections, testing, the treatment of infected horses, and transportation restrictions [15].

In endemic regions, most infected horses are asymptomatic; however, these animals remain infectious, underscoring the importance of surveillance [14,16]. Since chronic carriers do not exhibit clinical symptoms but still retain infectivity, monitoring them in endemic areas is crucial for assessing the transmission risk of equine piroplasms, especially *T. equi* [14,17]. Generally, in Xinjiang, the *T. equi* infection rate is higher than that of *B. caballi*, and infections with *T. equi* typically persist for life [2,10,11,12,18,19,20]. Xinjiang is one of China’s most important livestock regions and has a rapidly developing horse industry. The total horse inventory in Xinjiang in 2020 was 954,500, meaning Xinjiang had the most horses in China; this number is steadily rising. Xinjiang is also a hyperendemic area for *T. equi*. The *T. equi* infection rate in some areas of Xinjiang was 40.8% in 2014, 39.5% in 2018, and 23.8% in 2021 [19,21,22]. Therefore, continuous monitoring and effective control measures in Xinjiang are essential to manage the *T. equi* transmission risk.

The taxonomic classification of *T. equi* has been controversial since its discovery [2,23,24]. Although the pathogen was ultimately named *T. equi*, additional data are still needed to determine its final classification. Genotype differences not only affect the diagnostic results but also lead to different clinical manifestations and treatment outcomes [25,26,27,28,29].

The *18S* rRNA gene was employed as the primary marker for species-specific detection and broad-range genotyping, as it represents the most widely used genetic marker for phylogenetic analysis of piroplasms due to its conserved regions [30]. *EMA-1*, a major merozoite surface protein of *T. equi* that plays a crucial role in parasite recognition, adhesion, and invasion processes. Its immunogenic properties make it a promising vaccine candidate antigen, and a competitive ELISA based on *EMA-1* has been widely adopted for serological diagnosis. Furthermore, *EMA-1* serves as an excellent target for investigating intraspecific genetic variation within *T. equi* [7,23,29,31]. Mitochondria, as the core organelles for energy metabolism in eukaryotes, possess mitochondrial genomes that serve as ideal molecular markers for phylogenetic reconstruction due to their moderate evolutionary rate and lack of recombination, meaning that they can play a pivotal role in phylogenetic studies of apicomplexan parasites [32,33]. These mitochondrial genomes not only help resolve taxonomic controversies but also reveal cryptic diversity and speciation events [34]. Furthermore, mitochondrial functional research is equally critical for identifying potential drug targets and investigating resistance mechanisms [34,35]. The mitochondrial genome of *T. equi* exhibits a unique structural feature, demonstrating near-complete loss of synteny compared to species like *B. bigemina*, *B. caballi*, *B. gibsoni*, and *T. orientali* [36]. However, current phylogenetic studies on *T. equi* based on the mitochondrial genome remain limited [12], which significantly hindering population genetics research and the optimization of *T. equi* control strategies.

Some studies have demonstrated that the *18S* rRNA genotype A of *T. equi* isolates is more likely to induce clinical symptoms in infected horses [29,37]. Moreover, there are also some studies indicating that imidocarb dipropionate can eliminate infections caused by genotype A of *T. equi*, but not those caused by *T. haneyi* [25,27]. Variations in antigen expression or treatment sensitivity among different *T. equi* genotypes could lead to changes in accuracy or sensitivity during diagnosis [4,38]. However, research on *T. equi* genotypes has mainly focused on the *18S* rRNA and *EMA-1* genes, while there is relatively less research on mitochondrial genotypes [12]. Therefore, the objectives of the present study were to (1) investigate the prevalence of *T. equi* during the period 2023–2024, (2) further characterize the genotypic composition of this pathogen, and (3) analyze its haplotype diversity.

## 2. Materials and Methods

### 2.1. Blood Sample Collection and DNA Extraction

The sample collection began in May 2023 and finished in May 2024. A total of 440 equine blood samples were collected in Xinjiang, China. The study area has a temperate continental arid and semi-arid climate. The total sample size was calculated to estimate the prevalence of *T. equi* in Xinjiang with a 95% confidence level and a 5% margin of error, based on a previously reported prevalence of 38.9% in China [18]. None of the horses exhibited clinical symptoms at the time of sampling.

**Figure 1:**
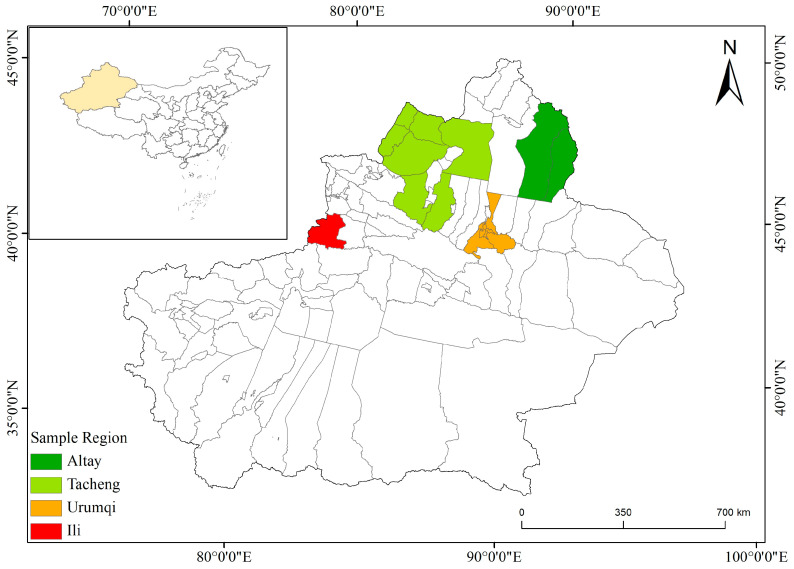
Map of the study area. Samples were collected from four regions: dark green represents Altay, light green represents Tacheng, red represents Ili, and green represents Urumqi.

Of the samples, 249 were collected in the Altay region, 88 were collected in the Ili region, 51 were collected in the Tacheng region, and 52 were collected in Urumqi (Figure 1). Approximately 5 mL of blood was collected from the external jugular vein of each horse into an EDTA-coated vacutainer tube. DNA was extracted using the TIANamp Blood DNA kit (TIANGEN, Beijing, China) according to the manufacturer’s instructions, and then stored at −20 °C until used. All animal protocols were reviewed by the Institutional Animal Care and Use Committee of Xinjiang Agricultural University (protocol number: GB/T 35892-2018) [39].

### 2.2. PCR Detection of T. equi

All 440 DNA samples underwent screening using *T. equi*-specific PCR assays. Briefly, the PCR assay for detecting *T. equi* was conducted with forward (5′-TCGAAGACGATCAGATACCGTCG-3′) and reverse primers (5′-TGCCTTAAACTTCCTTGCGAT-3′) targeting the *18S* ribosomal RNA gene, which produce a 392 bp product [40]. The PCR was performed in a 25 μL mixture consisting of 2 × Taq PCR MasterMix II (CoWin, Taizhou, China), 2 μL of template DNA, 5 pmol of each primer, and 6.5 μL DNase/RNase-free water. The mixture was heated for 10 min at 96 °C, followed by 40 cycles of 1 min at 96 °C, 1 min annealing at 60.5 °C, and 1 min extension at 72 °C, before a final extension for 10 min at 72 °C. The success of amplification was verified by electrophoresis on a 1% agarose gel.

### 2.3. PCR Detection and Sequencing of T. equi Genotypes

Samples positive for *T. equi* were further used to detect the *18S* rRNA, *EMA-1*, and *COI* genes. The PCR mixture was the same as that previously mentioned. The primer sequences and annealing temperature are shown in Table 1 [5,41,42]. The success of amplification was verified by electrophoresis on a 1% agarose gel. Positive PCR products were sequenced using Sanger technology (Sangon Biotech, Shanghai, China). The results obtained after sequencing were analyzed using the NCBI BLAST+2.17.0 algorithm, and the sequence data generated were submitted to GenBank under the following accession numbers: *18S* rRNA: PQ187782-PQ187820, *EMA-1*: PQ305366-PQ305398, *COI*: PQ305399-PQ305405.

### 2.4. Phylogenetic Analysis Based on Multi-Gene Sequences

Three phylogenetic trees were generated utilizing *18S* rRNA, *EMA-1*, and *COI* gene sequences. For the *18S* rRNA gene PCR products, 39 samples yielding clear and distinct bands were selected for sequencing; for the *EMA-1* gene PCR products, 33 such samples were chosen for sequencing; and for the *COI* gene PCR products, 7 samples with a single clear and distinct band were selected for sequencing. Of the 39 samples successfully sequenced for the *18S* rRNA gene, 26 were from Tacheng (PQ187795-PQ187820), 5 were from Ili (PQ187790-PQ187794), 5 were from Urumqi (PQ187782, PQ187786-PQ187789), and 3 were from Altay (PQ187783-PQ187785). For the *EMA-1* gene, of the 33 samples successfully sequenced, 9 were from Tacheng (PQ305366-PQ305374), 21 were from Urumqi (PQ305375-PQ305395), and 3 were from Altay (PQ305396-PQ305398). Only 7 samples were successfully sequenced for the *COI* gene, 3 from Tacheng (PQ305399-PQ305401) and 4 from Urumqi (PQ305402-PQ305405). Multiple sequence alignment of the obtained sequences was generated using Clustal W within MEGA 11 software, using a gap opening penalty of 15 and gap extension penalty of 6.66 for the pairwise and multiple alignments, respectively.

The Compute Nucleotide Composition function in MEGA 11 [43] was used to determine the nucleotide composition of the sequence. DnaSP 6 software [44] was used to calculate the number of haplotypes, haplotype diversity, and nucleotide diversity. Meanwhile, the haplotype network was constructed using PopART 1.7 software [45].

The Maximum Likelihood (ML) approach and the Kimura 2-parameter (K2P) model with 1000 bootstrap samplings were used to infer the evolutionary history using MEGA 11. In the construction of the *18S* rRNA phylogenetic tree, the *18S* rRNA gene sequences of *T. annulata* (DQ287944.1), *T. parva* (L02366.1), and *B*. *caballi* (EU888904.1) were used as the outgroup. In the construction of the *EMA-1* phylogenetic tree, the *EMA-2* (AB013725.1) and *EMA-3* (AB204709.1) genes of *T. equi*, as well as major piroplasm surface protein gene of *T. buffeli* (D78015.1), were used as the outgroup. In the construction of the *COI* phylogenetic tree, the *COI* (LC852773.1) of *T. annulata*, was used as the outgroup.

### 2.5. Statistical Analyses

The 95% confidence intervals (CIs) for prevalence rates were calculated using VassarStats (VassarStats: Statistical Computation Web Site) to estimate the potential ranges of true prevalence. Statistical significance (*p* values) was determined with SPSS 27 (SPSS Software, IBM, Armonk, NY, USA) [46]. To determine if the differences in infection rates among the four sampling regions (Altay, Ili, Tacheng, and Urumqi) were statistically significant, a Pearson’s chi-squared (χ^2^) test of independence was performed. *p*-values of less than 0.05 were considered to indicate statistical significance.

## 3. Results

### 3.1. Molecular Detection of T. equi

Among the 440 sampled horses, 169 were positive for *T. equi*, corresponding to an overall prevalence of 38.41% (95% CI: 33.87–43.15). Regionally, the prevalence varied significantly: 52 (20.88%), 44 (50.00%), 44 (86.27%), and 29 (55.77%) positive cases were detected in Altay, Ili, Tacheng, and Urumqi, respectively. Using the chi-squared test, we found statistically significant differences in infection rates between regions (*p* < 0.001). Further comparing the infection rates in different regions, we found that, except for the infection rates in Ili and Urumqi, which had no statistical differences (*p* = 0.461), the pairwise comparisons between other regions were statistically different (*p <* 0.001) (Table 2).

### 3.2. Phylogenetic Analyses

For *18S* rRNA genotyping, two distinct genotypes (A and E) were identified among the 39 sequenced samples. Thirty-seven (94.9%) sequences clustered within genotype E, while the remaining two (5.1%) sequences formed a separate clade representing genotype A. Within the genotype E clade, our sequences showed close phylogenetic affinity with *T. equi* isolates collected in diverse geographic regions, including Russia (MG551915), Sri Lanka (LC775898), South Korea (HM229407), Spain (DQ287951), and Paraguay. The two genotype A sequences from Urumqi clustered closely with reference strains from Chile (MT463609), Iran (MK615933), Turkey (MG569904), Paraguay (LC775901), Cuba (KY111762), Israel (KX227640), India (KP995259), United States (JX177673), South Africa (EU888902), Brazil (KY952226), Sri Lanka (LC775887), and Spain (AY150062).

**Figure 2:**
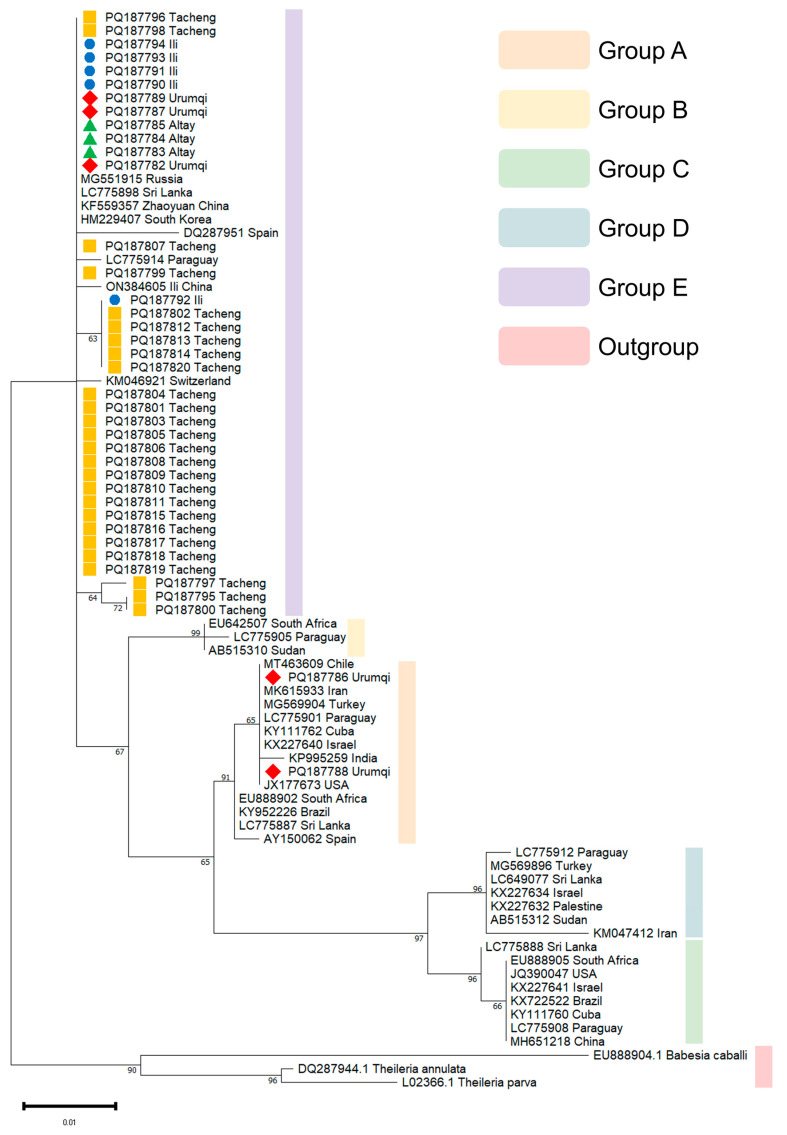
Phylogenetic assessment of various isolates of the *T. equi 18S* rRNA gene. Phylogenetic tree analysis was conducted using the Maximum Likelihood approach and the Kimura 2-parameter model with 1000 bootstrap samplings. Yellow squares represent samples from Tacheng, blue circles represent samples from Ili, red rhombi represent samples from Urumqi, and green triangles represent samples from Altay.

Geographically, all samples from Tacheng, Ili, and Altay belonged to genotype E, whereas Urumqi exhibited co-circulation of both genotypes, A (PQ187786, PQ187788) and E (PQ187789, PQ187787, PQ187782) (Figure 2).

**Figure 3:**
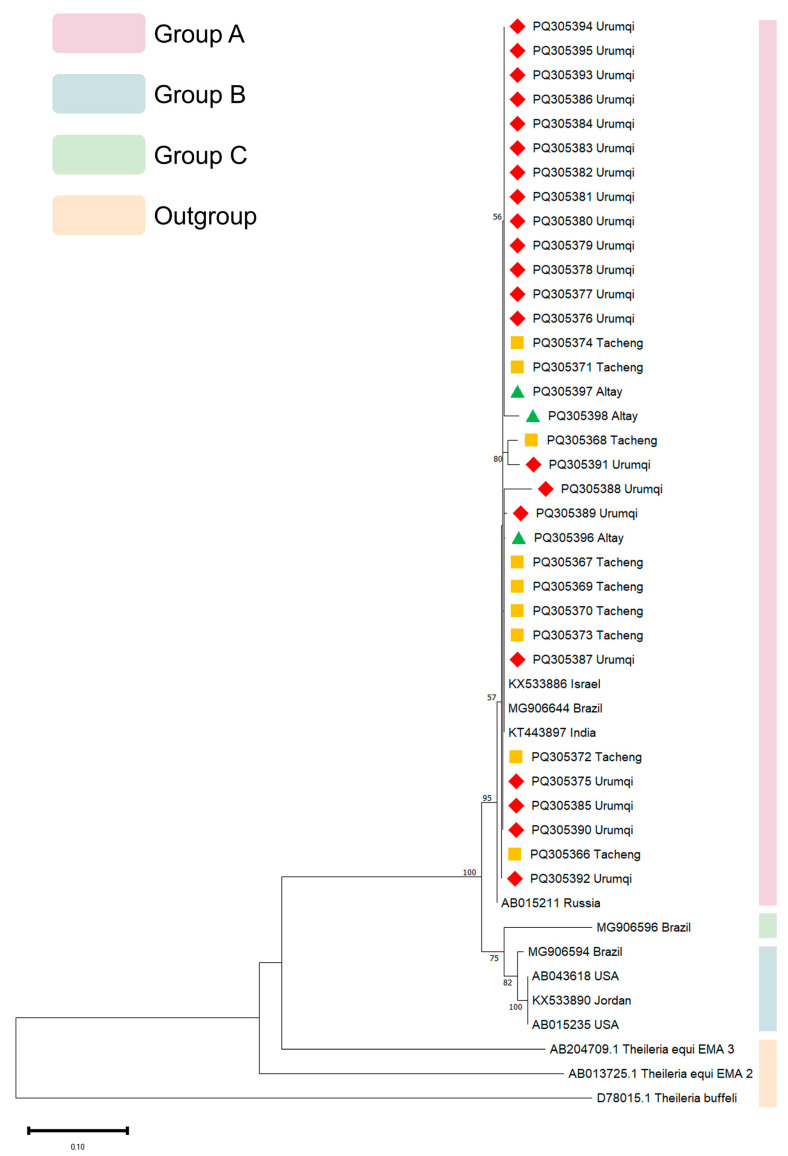
Phylogenetic analysis of various isolates of the *T. equi EMA-1* gene. Phylogenetic tree analysis was conducted using the Maximum Likelihood approach and the Kimura 2-parameter model with 1000 bootstrap samplings. Yellow squares represent samples from Tacheng, red rhombi represent samples from Urumqi, and green triangles represent samples from Altay.

All *EMA-1* gene sequences generated in this study clustered within genotype A, demonstrating close phylogenetic affinity to previously reported strains from Israel (KX533886), Brazil (MG906644), India (KT443897), and Russia (AB015211). Genotype B comprised a single sequence from Brazil (MG906596), while genotype C included two sequences from the USA (AB043618, AB015235), one from Brazil (MG906594), and one from Jordan (KX533890) (Figure 3).

**Figure 4:**
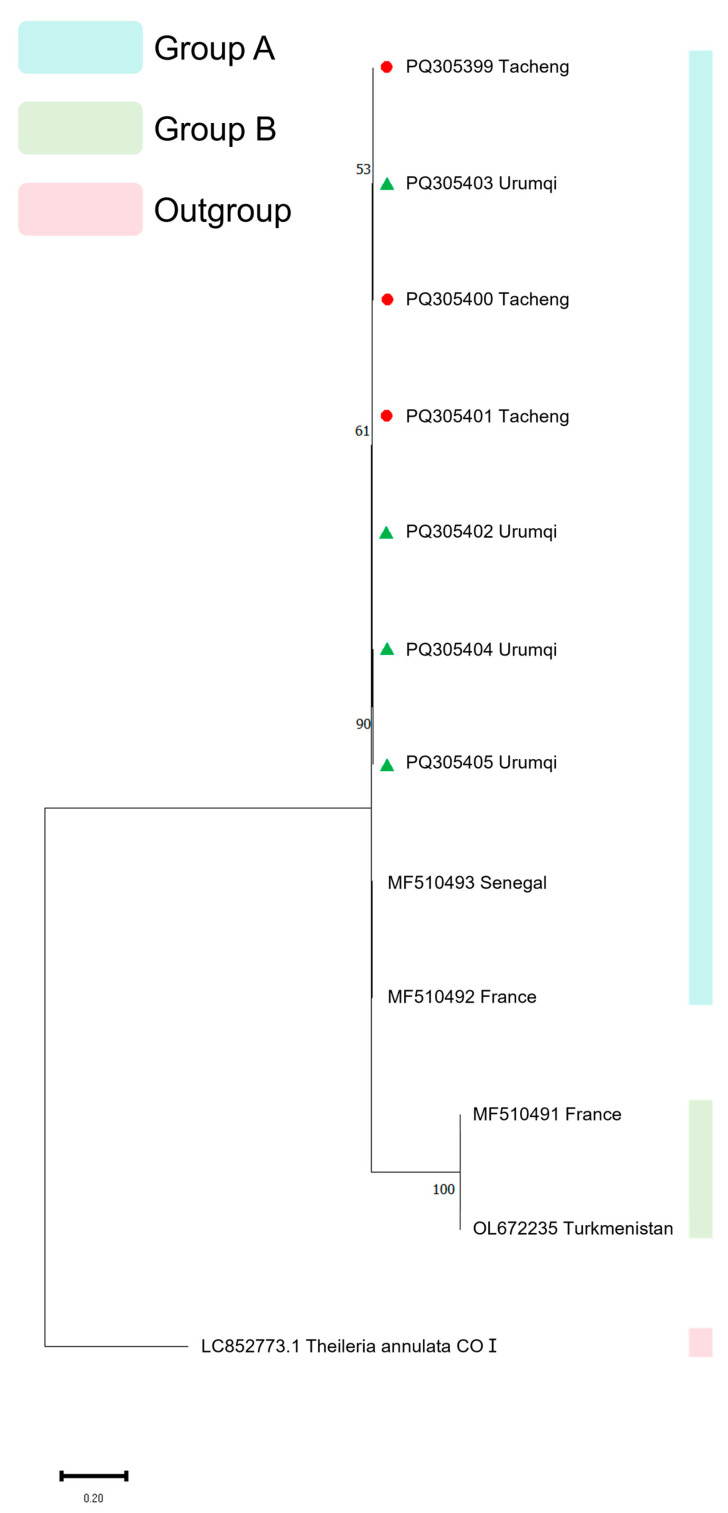
Phylogenetic analysis of various isolates of the *T. equi COI* gene. Phylogenetic tree analysis was conducted using the Maximum Likelihood approach and the Kimura 2-parameter model with 1000 bootstrap samplings. Red circles represent samples from Tacheng, and green triangles represent samples from Urumqi.

The phylogenetic tree analysis of the *COI* gene revealed that all seven sequenced samples clustered together in a single clade, exhibiting a closer evolutionary relationship with isolates from Senegal and France. Concurrently, another French isolate and a Turkmenistan isolate formed a distinct, independent clade (Figure 4).

### 3.3. Haplotype Analyses

#### 3.3.1. *18S* rRNA Gene

**Figure 5:**
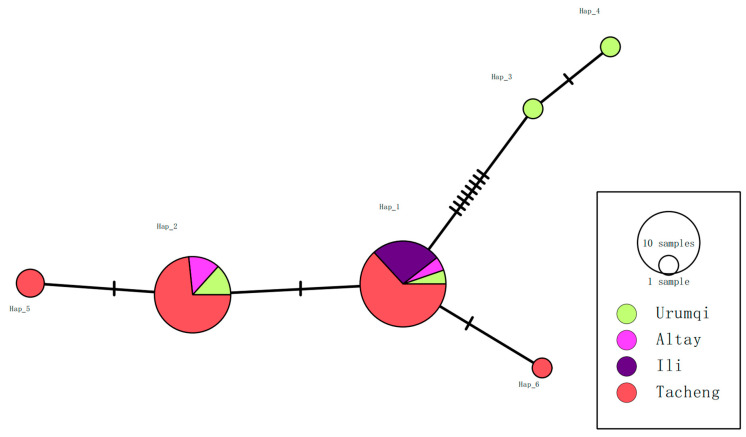
Haplotype TCS network of *Theileria equi 18S* rRNA sequences (333 bp) detected in horse blood samples from Xinjiang, China. Each color corresponds to a unique population. The size of each circle is proportional to the frequency of the respective haplotype. Each small dash represents a mutational event.

Among the 39 sequenced samples, six haplotypes were identified for the *18S* rRNA gene. Tacheng and Urumqi exhibited the highest haplotype richness (n = 4 each), while Altay and Ili had lower richness (n = 2 and n = 1, respectively). Haplotype diversity (Hd) varied markedly across regions: Ili had the lowest Hd (0.0000), reflecting a single fixed haplotype, while Urumqi displayed the highest Hd (0.9000). Nucleotide diversity (π) followed a similar trend and was highest in Urumqi (0.01747) (Table 3). Notably, Hap_1 was shared across all four regions, indicating a widespread ancestral lineage, whereas Hap_2 was restricted to Urumqi, Altay, and Tacheng (Figure 5).

#### 3.3.2. *EMA-1* Gene

**Figure 6:**
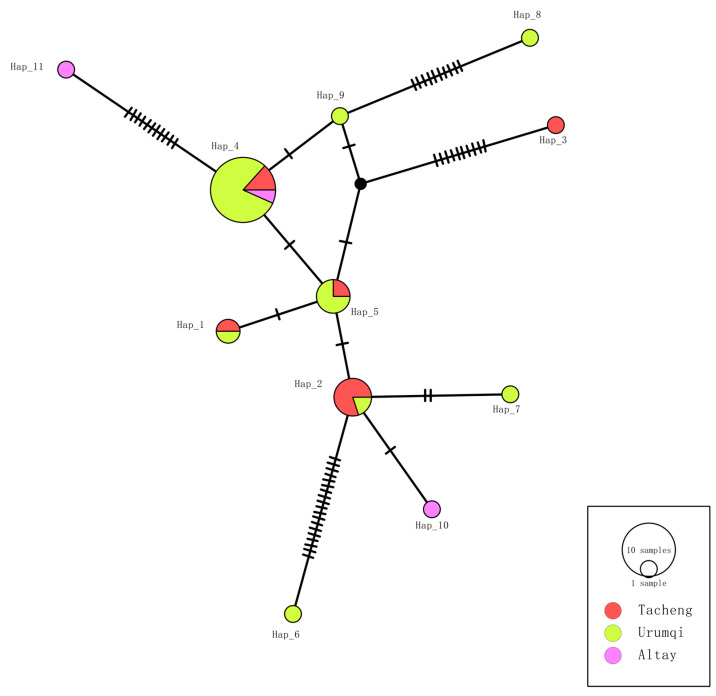
Haplotype TCS network of *Theileria equi EMA-1* sequences (564 bp) detected in horse blood samples from Xinjiang, China. Each color corresponds to a unique population. The size of each circle is proportional to the frequency of the respective haplotype. Each small dash represents a mutational event. The black circle indicates the median vector.

For the *EMA-1* gene, 11 haplotypes were identified among 33 samples. Urumqi had the highest number of haplotypes (n = 8), Altay exhibited the greatest haplotype diversity (Hd = 1.000) due to its smaller sample size (n = 3), and Urumqi showed the lowest diversity (Hd = 0.671). Nucleotide diversity was also highest in Altay (π = 0.01688) (Table 3), suggesting localized genetic drift or selection pressure. Haplotype sharing patterns revealed Hap_4 as unique to three regions, whereas Hap_1, Hap_2, and Hap_5 were shared between Tacheng and Urumqi (Figure 6).

#### 3.3.3. *COI* Gene

**Figure 7:**
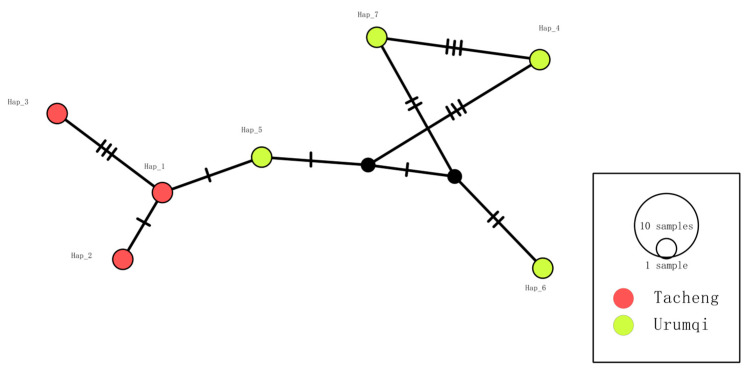
Haplotype TCS network of *Theileria equi COI* sequences (778 bp) detected in horse blood samples from Xinjiang, China. Each color corresponds to a unique population. The size of each circle is proportional to the frequency of the respective haplotype. Each small dash represents a mutational event. Black circles indicate median vectors.

*COI* gene analysis of the seven sequenced samples identified seven distinct haplotypes, with Tacheng and Urumqi harboring three and four haplotypes, respectively. Both regions showed high haplotype diversity (Hd = 1.0000), but nucleotide diversity was relatively low in Tacheng (π = 0.00344) (Table 3), indicating a recent population expansion or purifying selection. The haplotype network revealed that there were no shared haplotypes between Tacheng and Urumqi (Figure 7), suggesting limited gene flow or ecological isolation.

## 4. Discussion

This molecular epidemiological study analyzed 440 equine samples from four regions in Xinjiang (Altay, Ili, Tacheng, and Urumqi) collected between 2023 and 2024. The overall prevalence of *T. equi* infection was 38.41% (95% CI: 33.87–43.15), consistent with a previous report from China (38.9%. 2018–2020) [18]. However, this infection rate is substantially higher than the global estimated prevalence of 29.4% [47]. Current data indicate that the prevalence of *T. equi* infection in China is notably higher than in some Asian countries (1.3% in Thailand [48]) and similar to that in others (24.8% in Kyrgyzstan [14] and 20.79% in India [7]), but lower than the very high rates reported in Sri Lanka (85.6%) [49] and Mongolia (78.2%) [38]. The extremely high prevalence of infection in Sri Lanka and Mongolia may be attributed to ecological and husbandry factors that are highly favorable to the tick-parasite cycle, such as the optimal climatic conditions for vector ticks, extensive pastoral management systems with limited acaricide use, and potentially high densities of equine hosts. The stable overall prevalence of *T. equi* infection reflects the characteristics of an endemic disease.

The results demonstrated geographical variations in the prevalence of *T. equi* across Xinjiang, potentially associated with temperature, humidity, and rainfall patterns [50] and the distribution of tick vectors. Known tick vectors of *T. equi* include *Hyalomma excavatum*, *H. anatolicum*, *Rhipicephalus bursa*, *R. microplus*, and *Amblyomma cajennense*, which have been reported in multiple regions worldwide [17]. In addition, studies in China confirm transmission by *D. nuttalli*, *D. silvarum*, *D. niveus*, and *Rhipicephalus haemaphysaloides* [51]. Tick species show distinct regional patterns. In Tacheng, the main tick species are *H. asiaticum*, *H. marginatum*, *D. niveus*, *D. nuttalli*, *D. silvarum*, *D. marginatus*, and *Haemaphysalis punctata*. The main tick species in Urumqi are *H. asiaticum*, *H. anatolicum*, and *Ixodes persulcatus*. In Ili, the main tick species are *H. marginatum*, *H. dromedarii*, *D. silvarum*, *D. marginatus*, *H. asiaticum*, *H. punctata*, *R. turanicus*, and *I. persulcatus*. In Altay, the main tick species are *D. nuttalli*, *D. niveus*, *D. marginatus*, *D. silvarum*, and *H. punctata* [52,53,54,55]. Therefore, we hypothesize that regional prevalence differences correlate with tick vector competence. For instance, the prevalence of infection in Urumqi (55.77%) being higher than that in Altay (20.88%) may stem from the presence of *H. anatolicum*, a highly efficient vector, despite this region hosting fewer tick species.

When compared with historical data, the prevalence of *T. equi* infection showed an upward trend across all surveyed regions. In Ili, the rate increased to 50.00% in the present study from earlier reports [21], while Tacheng showed a marked rise from 66.70% in 2020 to 86.27% in the current study [18]. Similarly, the prevalence of infection in Urumqi had surged from 17.7% (2018–2020) to 55.77% [56], while the prevalence in Altay had increased from 13.3% in 2016 to 20.88% in the current study [57].

Given the high prevalence of *T. equi* in Xinjiang, elucidating the characteristics of its genetic diversity is essential for formulating targeted control strategies. From an evolutionary perspective, low-pathogenicity genotypes may have a transmission advantage [29], as demonstrated in *B. rossi* genotype–pathogenicity correlations [58]. Genetic diversity influences diagnostic sensitivity [29] and the selection of vaccine antigens [31]. In this study, *18S* rRNA genotype E was predominant (94.87%, 37/39), with only two genotype A cases identified (exclusively in Urumqi). The predominance of genotype E may reflect its Eurasian-wide distribution and asymptomatic phenotype, whereas genotype A is linked to clinical disease [12,50]. In China, genotypes A, B, C, and E of *T. equi 18S* rRNA gene have been reported. Genotype A is primarily distributed in Inner Mongolia, Tianjin, and Jilin; genotype B has been found in only one case in Jilin; genotype C is mainly distributed in Tianjin and Gansu; and E genotype has the broadest distribution, having been detected in multiple regions including Anhui, Hebei, Heilongjiang, Henan, Inner Mongolia, Jilin, Liaoning, Shandong, Sichuan, Tianjin, and Xinjiang [21,59,60,61]. In this study, we confirmed that genotype A is also present in Xinjiang, specifically in Urumqi, a region which harbors both A and E genotypes. This contrasts with Ili, Altay, and Tacheng, which contain genotype E exclusively. Notably, the genotype in the Ili region is consistent with our detection and sampling results from 2021 [21]. The study detected two genotypes (A and E) in Urumqi, whereas only a single genotype (E) was found in other regions. The genetic diversity of Urumqi may reflect its role as a transportation hub with frequent equine trade.

Despite extensive *T. equi* genotyping studies in China, most have focused on a single gene [18,20,59,61]. Notably, the combined utilization of *18S* rRNA and *EMA-1* genes can provide a more comprehensive solution for elucidating the genetic diversity of *T. equi* [7,29]. In this study, phylogenetic analysis of the *EMA-1* gene revealed that all isolates belonged to genotype A, representing the first confirmation that the *18S* rRNA genotype E/*EMA-1* genotype A combinatorial pattern predominates among *T. equi* populations in Xinjiang.

Due to the limited availability of *COI* reference sequences for *T. equi* and the absence of a standardized genotyping system, we provisionally categorized the *COI* sequences of *T. equi* into two groups based on the phylogenetic tree results. The first group includes all Xinjiang isolates sequenced in this study, along with isolates from France and Senegal. The second group consists of another French isolate and an isolate from Turkmenistan. With further research, we anticipate that the genetic and evolutionary data for the *COI* gene in *T. equi* will be refined and expanded to establish a more comprehensive genotyping framework.

Haplotype diversity refers to the degree of genetic heterogeneity in combinations of single nucleotide polymorphisms (SNPs) exhibiting linkage disequilibrium within specific chromosomal regions and serves as a core indicator for evaluating genetic variation within populations [62]. In the field of parasitology, haplotype studies hold significant value for elucidating drug resistance mechanisms, guiding drug target screening, and optimizing prevention and control strategies [63]. For instance, specific haplotypes of *Plasmodium* species can enhance transmission efficiency by modulating the specific binding of gametocyte surface proteins to mosquito midgut receptors [64]. However, global research on *T. equi* haplotypes remains extremely limited [65], and there are no reports in China to date. To our knowledge, this study represents the first haplotype analysis of three genes in *T. equi* populations from Xinjiang, China.

Haplotype analysis of the *18S* rRNA gene revealed six distinct haplotypes among 39 samples, with Tacheng and Urumqi exhibiting four haplotypes each. For the *EMA-1* gene, haplotype analysis of 33 samples identified eight haplotypes in Urumqi. Similarly, *COI* gene haplotype analysis of seven samples detected four haplotypes in Urumqi. When considering the combined haplotype and nucleotide diversity results across all three genes, Urumqi demonstrated the highest levels for both parameters. This multi-locus enrichment of genetic diversity suggests that Urumqi may serve as an evolutionary hotspot for *T. equi* within the Xinjiang region. The distribution of Hap-1 in the *18S* rRNA gene across all four regions and Hap-4 in the *EMA-1* gene across three regions suggests that these two haplotypes may represent ancestral lineages. Alternatively, these haplotypes might carry advantageous mutations that have been independently selected for and retained across multiple regions due to their adaptive benefits.

Limitations: This study has several limitations. First, as detailed in the methods, individual demographic data (e.g., precise age, breed) and formal clinical assessments were not collected. Therefore, our analysis cannot identify host-specific risk factors for infection, and the reported prevalence represents the infection rate within a broadly defined, apparently healthy adult horse population. Future studies incorporating detailed host metadata would be valuable for a more comprehensive risk analysis. The analysis of the mitochondrial *COI* gene, while pioneering in the context of *T. equi* in China, was constrained by a small final sample size (n = 7). This limitation precludes strong conclusions regarding mitochondrial diversity and lineage structure. The technical difficulties associated with amplifying this locus from field-derived DNA are acknowledged. Nonetheless, these data provide a critical first step by confirming amplification feasibility and yielding initial sequences to populate international databases. The observed phylogenetic clustering with isolates from France and Senegal offers a testable hypothesis for future larger-scale studies. Establishing a comprehensive *COI* genotyping system remains a future priority, for which this study provides foundational data.

This study investigated the molecular prevalence of *T. equi* across four regions in Xinjiang, China, and elucidating the genetic characteristics of *T. equi* within the region. The results revealed varying levels of infection prevalence across all the investigated regions, with significant geographical variations potentially associated with the diversity of tick species in different areas. Genetic analyses demonstrated that *18S* rRNA genotype E and *EMA-1* genotype A were predominant among *T. equi* isolates in Xinjiang. Furthermore, this study performed the first haplotype analysis of *T. equi* in China, revealing Urumqi as a genetic diversity hotspot for the species.

## Figures and Tables

**Table 1:** Primer sequences and thermal cycling.

Gene	Name	Primers (5′–3′)		Length
*18S* rRNA	*18s*-F	CGAAGACGATCAGATACCGTCG		430 bp
	*18s*-R	TGCCTTAAACTTCCTTGCGAT		
*EMA1*	*EMA-1*F	GCATCCATTGCCATTTCGAG		744 bp
	*EMA-1*R	TGCGCCATAGACGGAGAAGC		
*COI*	*COI*-F	GTGAYGTTGTTTTTCCAAG		750 bp
	*COI*-R	CCWGTTGTACCTCCAAYDAC		
Thermal cycling				
	Initial denaturation	Denaturation	Annealing	Extension
*18S* rRNA	96 °C, 10 min	96 °C, 1 min	56 °C, 1 min	72 °C, 1 min × 37 cycles
*EMA1*	95 °C, 6 min	94 °C, 45 s	68 °C, 45 s	72 °C, 45 s × 35 cycles
*COI*	95 °C, 15 min	95 °C, 1 min	55 °C, 30 s	72 °C, 1 min × 40 cycles

**Table 2:** PCR detection of *Theileria equi* in horses.

Region	Positives	Total	Prevalence(%)	95% Confidence Interval
Altay	52	249	20.88 ^a^	16.11–26.57
Ili	44	88	50.00 ^b^	39.23–60.77
Tacheng	44	51	86.27 ^c^	73.12–93.85
Urumqi	29	52	55.77 ^b^	41.42–69.27
Total	169	440	38.41	33.87–43.15

Different superscripts represent significant differences, while the same superscript indicates that there is no significant difference.

**Table 3:** Genetic diversity parameters of three *Theileria equi* genes.

Gene	Origin	Number of Sequences	Number of Haplotypes	Haplotype Diversity	Nucleotide Diversity
*18S* rRNA	Tacheng	26	4	0.62462	0.00224
	Ili	5	1	0.00000	0.00000
	Urumqi	5	4	0.90000	0.01747
	Altay	3	2	0.66667	0.00201
	Total	39	6	0.62618	0.00453
*EMA-1*	Tacheng	9	5	0.80556	0.00653
	Urumqi	21	8	0.67143	0.00712
	Altay	3	3	1.00000	0.01688
	Total	33	11	0.76894	0.00789
*COI*	Tacheng	3	3	1.00000	0.00344
	Urumqi	4	4	1.00000	0.00580
	Total	7	7	1.00000	0.00608

## Data Availability

The original contributions presented in this study are included in the article. Further inquiries can be directed to the corresponding authors.

## Abbreviations

The following abbreviations are used in this manuscript:

*T. equi*	*Theileria equi*
PCR	Polymerase Chain Reaction
*T. haneyi*	*Theileria haneyi*
*B. caballi*	*Babesia caballi*
*T. annulata*	*Theileria annulata*
*T. parva*	*Theileria parva*
*T. buffeli*	*Theileria buffeli*

## References

[1] Nugraha A.B., Cahyaningsih U., Amrozi A., Ridwan Y., Agungpriyono S., Taher D.M., Guswanto A., Gantuya S., Tayebwa D.S., Tuvshintulga B. (2018). Serological and molecular prevalence of equine piroplasmosis in Western Java, Indonesia. Vet. Parasitol. Reg. Stud. Rep..

[2] Wise L., Kappmeyer L., Mealey R., Knowles D. (2013). Review of equine piroplasmosis. J. Vet. Intern. Med..

[3] Tamzali Y. (2013). Equine piroplasmosis: An updated review. Equine Vet. Educ..

[4] Knowles D.P., Kappmeyer L.S., Haney D., Herndon D.R., Fry L.M., Munro J.B., Sears K., Ueti M.W., Wise L.N., Silva M. (2018). Discovery of a novel species, *Theileria haneyi* n. sp., infective to equids, highlights exceptional genomic diversity within the genus Theileria: Implications for apicomplexan parasite surveillance. Int. J. Parasitol..

[5] Kalantari M., Sharifiyazdi H., Ghaemi M., Ghane M., Nazifi S. (2022). *Theileria equi* in the horses of Iran: Molecular detection, genetic diversity, and hematological findings. Vet. Parasitol. Reg. Stud. Rep..

[6] Camacho A., Guitian F., Pallas E., Gestal J., Olmeda A., Habela M., Telford Iii S., Spielman A. (2005). *Theileria (Babesia) equi* and *Babesia caballi* infections in horses in Galicia, Spain. Trop. Anim. Health Prod..

[7] Maharana B.R., Ganguly A., Potliya S., Kumar B., Singh H., Dash A., Khanna S. (2024). Molecular detection and characterization of prevailing *Theileria equi* genotype in equine from northern India. Res. Vet. Sci..

[8] Knowles D., Kappmeyer L., Stiller D., Hennager S., Perryman L. (1992). Antibody to a recombinant merozoite protein epitope identifies horses infected with *Babesia equi*. J. Clin. Microbiol..

[9] Uilenberg G. (2006). Babesia—A historical overview. Vet. Parasitol..

[10] de Waal D.T. (1992). Equine piroplasmosis: A review. Br. Vet. J..

[11] Rothschild C.M. (2013). Equine piroplasmosis. J. Equine Vet. Sci..

[12] Tirosh-Levy S., Gottlieb Y., Fry L.M., Knowles D.P., Steinman A. (2020). Twenty Years of Equine Piroplasmosis Research: Global Distribution, Molecular Diagnosis, and Phylogeny. Pathogens.

[13] Sears K.P., Kappmeyer L.S., Wise L.N., Silva M., Ueti M.W., White S., Reif K.E., Knowles D.P. (2019). Infection dynamics of *Theileria equi* and *Theileria haneyi*, a newly discovered apicomplexan of the horse. Vet. Parasitol..

[14] Atabek B., Zhyldyz A., Aitakin K., Rysbek N., Jailobek O., Ahedor B., Mumbi N.N.M., Ma Y., Otgonsuren D., Perera W. (2024). Molecular prevalence and genotypic diversity of *Theileria equi* and *Babesia caballi* infecting horses in Kyrgyzstan. Parasitol. Int..

[15] Traub-Dargatz J., Bischoff B., James A., Freier J. (2010). A Literature Review of Equine Piroplasmosis. https://www.cabidigitallibrary.org/doi/pdf/10.5555/20113188077#core-collateral-purchase-access.

[16] Ahedor B., Sivakumar T., Valinotti M.F.R., Otgonsuren D., Yokoyama N., Acosta T.J. (2023). PCR detection of *Theileria equi* and *Babesia caballi* in apparently healthy horses in Paraguay. Vet. Parasitol. Reg. Stud. Rep..

[17] Onyiche T.E., Suganuma K., Igarashi I., Yokoyama N., Xuan X., Thekisoe O. (2019). A review on equine piroplasmosis: Epidemiology, vector ecology, risk factors, host immunity, diagnosis and control. Int. J. Environ. Res. Public Health.

[18] Wu J., Cui Y., Yu F., Muhatai G., Tao D., Zhao A., Ning C., Qi M. (2023). Prevalence and genetic characterization of *Theileria equi* and *Babesia caballi* in grazing horses in Xinjiang, northwestern China. Parasitol. Res..

[19] Li J., Li Y., Moumouni P.F.A., Lee S.H., Galon E.M., Tumwebaze M.A., Yang H., Huercha, Liu M., Guo H. (2020). First description of *Coxiella burnetii* and *Rickettsia* spp. infection and molecular detection of piroplasma co-infecting horses in Xinjiang Uygur Autonomous Region, China. Parasitol. Int..

[20] Cui Y., Cao M., Yu F., Zhao A., Tao D., Zhu T., Zhang Z., Qi M. (2024). Molecular detection of piroplasms in domestic donkeys in Xinjiang, China. Vet. Med. Sci..

[21] Zhang Y., Shi Q., Laven R., Li C., He W., Zheng H., Liu S., Lu M., Yang D.A., Guo Q. (2023). Prevalence and genetic diversity of *Theileria equi* from horses in Xinjiang Uygur Autonomous region, China. Ticks Tick-Borne Dis..

[22] Zhang Y., Chahan B., Liu S., Song R., Li Y., Guo Q., Wu H., Zhu Y. (2017). Epidemiologic studies on *Theileria equi* infections for grazing horses in Ili of Xinjiang province. Vet. Parasitol..

[23] Kappmeyer L.S., Thiagarajan M., Herndon D.R., Ramsay J.D., Caler E., Djikeng A., Gillespie J.J., Lau A.O., Roalson E.H., Silva J.C. (2012). Comparative genomic analysis and phylogenetic position of *Theileria equi*. BMC Genom..

[24] Mehlhorn H., Schein E. (1998). Redescription of *Babesia equi* Laveran, 1901 as *Theileria equi* Mehlhorn, Schein 1998. Parasitol. Res..

[25] Ahedor B., Otgonsuren D., Zhyldyz A., Guswanto A., Ngigi N.M.M., Valinotti M.F.R., Kothalawala H., Kalaichelvan N., Silva S.S.P., Kothalawala H. (2023). Development and evaluation of specific polymerase chain reaction assays for detecting *Theileria equi* genotypes. Parasites Vectors.

[26] Ueti M.W., Mealey R.H., Kappmeyer L.S., White S.N., Kumpula-McWhirter N., Pelzel A.M., Grause J.F., Bunn T.O., Schwartz A., Traub-Dargatz J.L. (2012). Re-emergence of the apicomplexan *Theileria equi* in the United States: Elimination of persistent infection and transmission risk. PLoS ONE.

[27] Sears K., Knowles D., Dinkel K., Mshelia P.W., Onzere C., Silva M., Fry L. (2020). Imidocarb dipropionate lacks efficacy against *Theileria haneyi* and fails to consistently clear *Theileria equi* in horses co-infected with *T. haneyi*. Pathogens.

[28] Bhoora R., Quan M., Matjila P.T., Zweygarth E., Guthrie A.J., Collins N.E. (2010). Sequence heterogeneity in the equi merozoite antigen gene (ema-1) of *Theileria equi* and development of an ema-1-specific TaqMan MGB™ assay for the detection of *T. equi*. Vet. Parasitol..

[29] Manna G., Cersini A., Nardini R., Del Pino L.E.B., Antognetti V., Zini M., Conti R., Lorenzetti R., Veneziano V., Autorino G.L. (2018). Genetic diversity of *Theileria equi* and *Babesia caballi* infecting horses of Central-Southern Italy and preliminary results of its correlation with clinical and serological status. Ticks Tick-Borne Dis..

[30] Kumar B., Maharana B.R., Thakre B., Brahmbhatt N.N., Joseph J.P. (2022). 18S rRNA gene-based piroplasmid PCR: An assay for rapid and precise molecular screening of Theileria and Babesia species in animals. Acta Parasitol..

[31] Munkhjargal T., Sivakumar T., Battsetseg B., Nyamjargal T., Aboulaila M., Purevtseren B., Bayarsaikhan D., Byambaa B., Terkawi M.A., Yokoyama N. (2013). Prevalence and genetic diversity of equine piroplasms in Tov province, Mongolia. Infect. Genet. Evol..

[32] Gray M.W., Lang B.F., Burger G. (2004). Mitochondria of protists. Annu. Rev. Genet..

[33] Yang X., Tang S., Du C., Chen Y., Luo Z., Li M., Liu S., Duan M., Jiang D., Shen Y. (2025). Insights into the mitochondrial genome structure and phylogenetic placement of *Theileria velifera* in comparison to other apicomplexan parasites. Sci. Rep..

[34] Ulucesme M.C., Aktas M., Ozubek S. (2024). Mitochondrial Genome Analysis of *Babesia ovis* (Apicomplexa: Babesiidae) Endemic in Sheep in Türkiye. Vet. Sci..

[35] Birth D., Kao W.C., Hunte C. (2014). Structural analysis of atovaquone-inhibited cytochrome bc1 complex reveals the molecular basis of antimalarial drug action. Nat. Commun..

[36] Hikosaka K., Watanabe Y., Tsuji N., Kita K., Kishine H., Arisue N., Palacpac N.M., Kawazu S., Sawai H., Horii T. (2010). Divergence of the mitochondrial genome structure in the apicomplexan parasites, Babesia and Theileria. Mol. Biol. Evol..

[37] Hall C.M., Busch J.D., Scoles G.A., Palma-Cagle K.A., Ueti M.W., Kappmeyer L.S., Wagner D.M. (2013). Genetic characterization of *Theileria equi* infecting horses in North America: Evidence for a limited source of U.S. introductions. Parasit. Vectors.

[38] Otgonsuren D., Amgalanbaatar T., Narantsatsral S., Enkhtaivan B., Munkhgerel D., Zoljargal M., Davkharbayar B., Myagmarsuren P., Battur B., Battsetseg B. (2024). Epidemiology and genetic diversity of *Theileria equi* and *Babesia caballi* in Mongolian horses. Infect. Genet. Evol..

[39] Committee S.A. (2018). General Administration of Quality Supervision, Inspection and Quarantine of the People’s Republic of China & Standardization Administration of China.

[40] Alhassan A., Pumidonming W., Okamura M., Hirata H., Battsetseg B., Fujisaki K., Yokoyama N., Igarashi I. (2005). Development of a single-round and multiplex PCR method for the simultaneous detection of *Babesia caballi* and *Babesia equi* in horse blood. Vet. Parasitol..

[41] Kumar S., Sudan V., Shanker D., Devi A. (2020). *Babesia (Theileria) equi* genotype A among Indian equine population. Vet. Parasitol. Reg. Stud. Rep..

[42] Dahmana H., Amanzougaghene N., Davoust B., Normand T., Carette O., Demoncheaux J.P., Mulot B., Fabrizy B., Scandola P., Chik M. (2019). Great diversity of Piroplasmida in Equidae in Africa and Europe, including potential new species. Vet. Parasitol. Reg. Stud. Rep..

[43] Tamura K., Stecher G., Kumar S. (2021). MEGA11: Molecular evolutionary genetics analysis version 11. Mol. Biol. Evol..

[44] Rozas J., Ferrer-Mata A., Sánchez-DelBarrio J.C., Guirao-Rico S., Librado P., Ramos-Onsins S.E., Sánchez-Gracia A. (2017). DnaSP 6: DNA sequence polymorphism analysis of large data sets. Mol. Biol. Evol..

[45] Leigh J.W., Bryant D., Nakagawa S. (2015). POPART: Full-feature software for haplotype network construction. Methods Ecol. Evol..

[46] Salcedo J., McCormick K. (2020). SPSS Statistics for Dummies.

[47] Onyiche T.E., Taioe M.O., Molefe N.I., Biu A.A., Luka J., Omeh I.J., Yokoyama N., Thekisoe O. (2020). Equine piroplasmosis: An insight into global exposure of equids from 1990 to 2019 by systematic review and meta-analysis. Parasitology.

[48] Phetkarl T., Fungwithaya P., Lewchalermvong K., Sontigun N. (2024). Prevalence of gastrointestinal and blood parasites in horses of Nakhon Si Thammarat province, Thailand. Vet. World.

[49] Ahedor B., Kothalawala H., Kanagaratnam R., Vimalakumar S.C., Otgonsuren D., Tuvshintulga B., Batmagnai E., Silva S.S.P., Sivakumar T., Yokoyama N. (2022). First detection of *Theileria equi* in free-roaming donkeys (*Equus africanus asinus*) in Sri Lanka. Infect. Genet. Evol..

[50] Jouglin M., Bonsergent C., de la Cotte N., Mège M., Bizon C., Couroucé A., Lallemand É.A., Leblond A., Lemonnier L.C., Leroux A. (2025). Equine piroplasmosis in different geographical areas in France: Prevalence heterogeneity of asymptomatic carriers and low genetic diversity of *Theileria equi* and *Babesia caballi*. Ticks Tick. Borne Dis..

[51] Fanyao K. (2010). Livestock Parasitology.

[52] Liu Z. (2019). Geographical Distribution and Molecular Characteristics of Ticks and Molecular Detection of Important Tick-borne Pathogens in Northern Xinjiang. Ph.D. Thesis.

[53] Tang L., Wang Y., Liu D., Bu S. (2022). Tick Distribution in Xinjiang and Research Progress of Tick-Borne Diseases. Chin. J. Anim. Infect. Dis..

[54] Zhang L. (2014). Study on Geographical Distribution and Detection Pathogeny of Ticks. Master’s Thesis.

[55] Wang B. (2016). Species Identification, Phylogenetic Analysis of Hyalomma Species and Molecular Detection of Theileriosis Carried by Hyalomma, Xinjiang. Master’s Thesis.

[56] Peipei X. (2020). Prokaryotic Expression of RAP-1 Protein of Theileria equi and Its Effect on PBMC of Horse.

[57] Yutao Z., Qiabudan A., Ruiqi S., Bingjie W., Tuerxun, Muheyati S., Bayinchahan (2016). Preliminary report on detection of *Babesia caballi* and *Theileria equi* antibodies in herding horses in Fuyun county of Altay. Anim. Husb. Vet. Med..

[58] Matjila P., Carcy B., Leisewitz A., Schetters T., Jongejan F., Gorenflot A., Penzhorn B. (2009). Preliminary evaluation of the Br EMA1 gene as a tool for associating *Babesia rossi* genotypes and clinical manifestation of canine babesiosis. J. Clin. Microbiol..

[59] Zhao S., Wang H., Zhang S., Xie S., Li H., Zhang X., Jia L. (2020). First report of genetic diversity and risk factor analysis of equine piroplasm infection in equids in Jilin, China. Parasit. Vectors.

[60] Chen K., Hu Z., Yang G., Guo W., Qi T., Liu D., Wang Y., Du C., Wang X. (2022). Development of a duplex real-time PCR assay for simultaneous detection and differentiation of *Theileria equi* and *Babesia caballi*. Transbound. Emerg. Dis..

[61] Wang J., Liu J., Yang J., Wang X., Li Z., Xu J., Li X., Xiang Q., Li Y., Liu Z. (2019). The first molecular detection and genetic diversity of *Babesia caballi* and *Theileria equi* in horses of Gansu province, China. Ticks Tick. Borne Dis..

[62] Liang Z.Q., Han Y.F., Zeng G.P., Liang J.D., Chen W.H., Zhang Z.Y., Dong C.B., Shao Q.Y. (2020). Haplotype and its application in fungal research. Mycosystema.

[63] Nkhoma S.C., Ahmed A.O.A., Zaman S., Porier D., Baker Z., Stedman T.T. (2021). Dissection of haplotype-specific drug response phenotypes in multiclonal malaria isolates. Int. J. Parasitol. Drugs Drug Resist..

[64] Onyango S.A., Machani M.G., Ochwedo K.O., Oriango R.M., Lee M.C., Kokwaro E., Afrane Y.A., Githeko A.K., Zhong D., Yan G. (2025). Plasmodium falciparum Pfs47 haplotype compatibility to *Anopheles gambiae* in Kisumu, a malaria-endemic region of Kenya. Sci. Rep..

[65] Torres R., Hurtado C., Pérez-Macchi S., Bittencourt P., Freschi C., de Mello V.V.C., Machado R.Z., André M.R., Müller A. (2021). Occurrence and Genetic Diversity of *Babesia caballi* and *Theileria equi* in Chilean Thoroughbred Racing Horses. Pathogens.

